# Bactericidal Activity of Copper-Zinc Hybrid Nanoparticles on Copper-Tolerant *Xanthomonas perforans*

**DOI:** 10.1038/s41598-019-56419-6

**Published:** 2019-12-27

**Authors:** Renato Carvalho, Kamil Duman, Jeffrey B. Jones, Mathews L. Paret

**Affiliations:** 1 University of Florida, North Florida Research and Education Center, Quincy, FL 32351 USA; 2 Plant Protection Central Research Institute, Ankara, Turkey; 3 Plant Pathology Department, University of Florida, Gainesville, FL USA

**Keywords:** Antimicrobial resistance, Pathogens

## Abstract

Bacterial spot of tomato, caused by *Xanthomonas perforans*, *X. euvesicatoria*, *X. vesicatoria* and *X. gardneri*, is a major disease, contributing to significant yield losses worldwide. Over dependence of conventional copper bactericides over the last decades has led to the prevalence of copper-tolerant strains of *Xanthomonas* spp., making copper bactericides ineffective. Thus, there is a critical need to develop new strategies for better management of copper-tolerant *Xanthomonas* spp. In this study, we investigated the antimicrobial activity of a hybrid nanoparticle, copper-zinc (Cu/Zn), on copper-tolerant and sensitive strains. The hybrid nanoparticle significantly reduced bacterial growth *in vitro* compared to the non-treated and micron-size commercial copper controls. Tomato transplants treated with the hybrid nanoparticle had significantly reduced disease severity compared to the controls, and no phytotoxicity was observed on plants. We also studied the hybrid nanoparticle effect on the bacterial pigment xanthomonadin using Near-Infra Red Raman spectroscopy as an indicator of bacterial degradation. The hybrid nanoparticle significantly affected the ability of *X. perforans* in its production of xanthomonadin when compared with samples treated with micron-size copper or untreated. This study sheds new light on the potential utilization of this novel multi-site Cu/Zn hybrid nanoparticle for bacterial spot management.

## Introduction

Tomatoes (*Solanum lycopersicum*) are one of the most important among vegetable crops worldwide^1^. Globally, tomato production account for around 170 million tons annually^1^. In the United States, fresh market and processed tomatoes together account for more than $1.8 billion^2^. Among the many diseases affecting tomatoes, bacterial spot, caused by four *Xanthomonas* spp., *X. perforans*, *X. vesicatoria, X. euvesicatoria*, and *X. gardneri*, is one of most devastating disease and occurs in tropical, subtropical, and temperate regions across the globe^3^. Under favorable environmental conditions, such as high temperature and rainfall, bacterial spot is almost impossible to manage and cause extensive leaf and fruit lesions, necrotic leaves, and defoliation^3–5^. The disease can cause a 10–50% yield reduction depending on its severity^5–7^. Ideal conditions for disease occurrence and spread are especially true in tropical and sub-tropical regions of the world including Florida, the leading US state in fresh market tomato production^2,8,9^, where climatic conditions of high humidity during the two growing seasons in the year that favor the disease occurrence and spread.

Prevention strategies, such as clean seed, weed control, sanitation of equipment, and the use of tolerant varieties while important, has only proven to be minimally effective, especially in tropical and subtropical regions^9^. In addition, despite providing only partial disease management, copper bactericides continue to be the grower standard for disease management due to minimal availability of alternatives. To improve upon efficacy, copper is routinely combined with ethylene-bis-dithiocarbamates (EBDC) fungicides, commonly known as mancozeb or maneb^9–11^. Unfortunately, due to the dependence on copper over the past many decades, bacterial spot pathogens have developed copper tolerance, making copper bactericides ineffective^12^. Tomato bacterial spot causal agents are increasingly being isolated throughout the world^3^. For example, most of the *Xanthomonas* spp. that cause bacterial spot in the Caribbean and Central America are confirmed copper-tolerant^13^. The same was confirmed in Ethiopia^14^ and Australia where tolerant strains resulted in a poor disease control using copper-based chemicals^15^. In Taiwan, Asia, isolated strains of *Xanthomonas* spp. are currently confirmed copper-tolerant^16^. In North America, Canada recently confirmed the outbreak of copper-tolerant strains of *Xanthomonas* spp^17^. In recent and prior studies in Florida, USA, *X. perforans* strains were found to be Cu tolerant, thus creating challenges for growers to effectively control bacterial spot of tomato^8–12^.

Due to the presence of Cu tolerant strains in Florida, many studies have been conducted to identify new approaches in managing copper-tolerant strains. Two promising approaches include: bacteriophages and systemic acquired resistance (SAR) inducers^18,19^. In the case of bacteriophages, it shows significant disease reduction compared to non-treated controls^18^. However, there are many challenges to overcome to make phage therapy more reliable, such as improving viability and persistence under field conditions^18,19^. Another approach has been to utilize synthetic SAR inducers, such as Acibenzolar S-Methyl (ASM; Actigard^®^ 50 WG; Syngenta, Greensboro, NC), which is commercially used as a copper alternative. ASM works by activating plant defense systems to increase transcription of pathogenesis-related genes. Although it is reliable under field conditions, disease control with ASM has not been associated with improved yield^19–21^. Given the limitations of bacteriophages and ASM as control strategies, there has been a need to identify other novel strategies.

In recent years, considerable interest has also been placed on nanoparticles to control phytopathogens. Compared to micron-size particles, they are uniquely different in terms of density, reactivity, stability, malleability, and antimicrobial activity^22^. Various antibacterial nanoparticles have been developed and studied for disease management of copper-tolerant *Xanthomonas* spp.^8,23–26^. For example, the use of silver (Ag) nanoparticles merged in a dsDNA-graphene oxide matrix (Ag-dsDNA-GO). Under greenhouse conditions, Ag-dsDNA-GO displayed high antibacterial properties, even with Ag concentrations as low as 13 ppm^24,25^. Although it shows great promise, processing of both Ag and dsDNA are cost prohibitive, possibly limiting its use. Another example is the development of three copper composites (copper-core shell silica, copper-multivalent and copper-fixed quat) that were shown to be effective *in vitro*, and unlike Ag-dsDNA-GO, they were cheaper and prepared using industrial grade nanoparticles^8^. Moreover, magnesium oxide nanoparticles were found to effectively reduce disease severity of bacterial spot under both greenhouse and field conditions^26^. In the same study, it was shown nano sized particles of Cu_2_O had bactericidal activity against a copper-tolerant *Xanthomonas* spp. strain, while micron sized copper hydroxide did not had bactericidal activity at the same concentration of Cu when tested *in vitro*.

In another study nano-formulated ZnO was tested for control of citrus canker, caused by copper-sensitive *X. citri* subsp. *citri*^27^. ZnO reduced the development of leaf lesions in green house tests on grapefruits. In the field, it performed better than micron-sized copper bactericide control for disease management. A copper silica gel coating and zinc oxide element (ZnO-nCuSi) was also shown in a recent study to have efficacy against citrus canker^28^. More tests using zinc against strains of *X. campestris* pv. *vesicatoria*, the causal agent of bacterial spot on pepper, showed tolerance to copper, but sensitive to Zn, confirming its efficacy in bacterial control^29^. Therefore, novel alternatives, consisting of nanoparticles of Ti, Cu, Ag, Mg and Zn, may hold novel antibacterial properties that are the same as, if not better to, commercial copper bactericides.

While independently these elements in the nanoparticle form were shown to have activity against *X. perforans*, a key question remains if we could improve efficacy of copper if used as a hybrid with other elements. Improvements are not only interesting to increase the antibacterial properties but also aimed at reducing the likelihood for development of resistance by the bacteria.

Nanoparticles tested over the last two decades have been shown to inhibit the bacterial growth rate; inactivate the potential of organism to produce crucial compounds; exert bactericidal rather than bacteriostatic activity; or interact with important bacterial structures, leading to deformation of the cell membrane followed by penetration, thus causing damage to the cell and eventual death^29,30^. However, the mechanism of antimicrobial activity of nanoparticles on *X. perforans* is not well understood on components of the bacterial cell. Raman spectroscopy is a tool used to identify and characterize molecules based on the inelastic scattering of light where photons loose or gain energy during vibrational transition. This provides a unique pattern, or fingerprint for an individual molecule and is associated with the vibration of a particular chemical bond or a single functional group^31^. In recent years, advancements in sample preparation and equipment functionality, as well as ever-increasing libraries of biological molecules, has made Raman spectroscopy a useful and critical tool for characterizing biological materials^31–33^. Raman fingerprinting of all major bacterial pathogens has been generated using Near Infra-Red (NIR) Laser -Raman Spectroscopy and Green Laser Resonance Raman Spectroscopy, which provides a detailed view of key molecules in the bacterial cell^34^. In previous studies, Raman spectroscopy was used to elucidate biochemical changes in the bacterial wilt of tomato pathogen, *Ralstonia solanacearum*, upon exposure to essential plant essential oils^35^. Key functional changes occurred in the presence of molecules indicated by shift in wavelength, known as Raman shift cm^−1^, and by intensity of the peaks of the molecules have been documented^35^. One of the key molecules and unique Raman fingerprints for *Xanthomonas* spp. is xanthomonadin, a brominated, aryl-polyene, yellow, water-insoluble pigment that are localized exclusively in the outer membrane of the bacterial cell. The pigment has been identified in numerous strains/pathovars of *Xanthomonas* in prior research^36,37^.

The effectiveness of Cu/Zn hybrid nanoparticles against extremely destructive copper-tolerant bacterial strains has not been reported before. Based on prior literature, we hypothesize that Cu/Zn, consisting of two metal elements can be a potential material with high antibacterial properties against copper-tolerant *X. perforans* strains. The element cannot only reduce the bacterial spot severity in tomatoes but also will affect production of the *Xanthomonas*-specific pigment, xanthomonadin. The objectives of this study were to 1) evaluate the antibacterial activity of Cu/Zn hybrid nanoparticles on copper-tolerant and copper-sensitive strains of *X. perforans*, 2) evaluate efficacy of Cu/Zn in reducing bacterial spot disease severity and risks associated with occurrence of phytotoxicity, and 3) elucidate biochemical changes in xanthomonadin in the bacterial cell following Cu/Zn hybrid nanoparticle exposure.

## Results

### The antibacterial action of Cu/Zn hybrid nanoparticles against copper-tolerant *Xanthomonas perforans in vitro*

**Figure 1:**
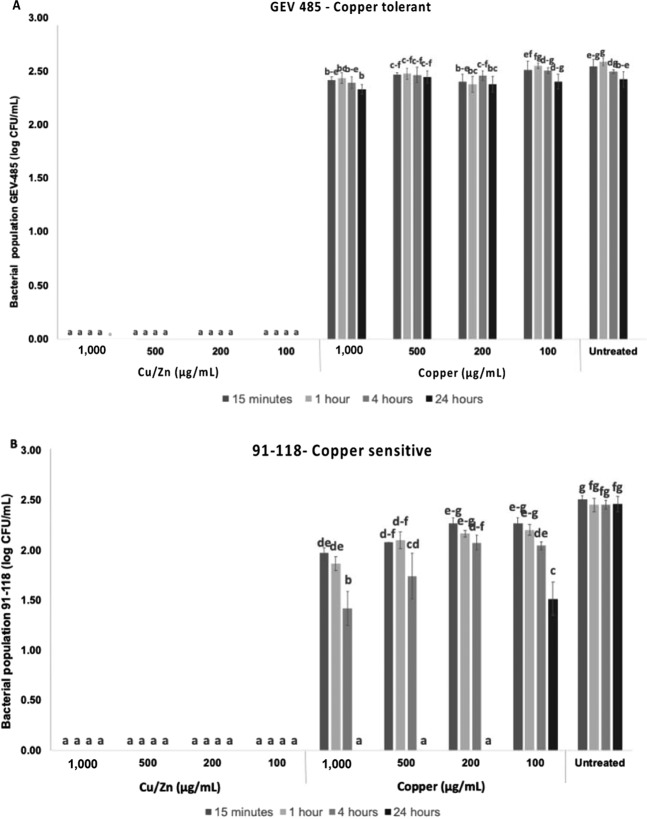
*In vitro* activity of Cu/Zn hybrid nanoparticles on *X. perforans* population at 15 min, 1 h, 4 h and 24 h. The experiment consisted of three replicates per treatment. The Cu/Zn hybrid nanoparticle treatments were 1000, 500, 200 and 100 µg/mL. Micron-sized commercial copper at the same concentrations; and sterile tap water served as the control. (**A**) Copper-tolerant *X. perforans* GEV485. (**B**) Copper-sensitive *X. perforans* 91–118. Error bars represent standard derivation. Statistical significance was based on a *P-value* < 0.05. *In vitro* experiments were conducted twice, and the effect of the experiment was not significant.

Cu/Zn hybrid nanoparticles at all concentrations had high antibacterial activity against copper-tolerant and sensitive strains (Fig. 1). When applied to the copper-tolerant strain, all concentrations of Cu/Zn hybrid nanoparticles completely inhibited bacterial growth in the tested exposure periods (Fig. 1A). None of micron-size commercial copper treatments (Kocide 3000) significantly reduced bacterial growth when compared to the untreated samples (P = 0.05, SNK). When applied to the copper-sensitive strain (91–118), Cu/Zn hybrid nanoparticles also performed similarly (Fig. 1B) inhibiting all the bacterial growth in the tested concentrations at all the exposure periods. The micron-sized commercial copper treatments at all concentrations and exposure durations of 15 min, 1 h and 4 h had statistically higher bacterial growth compared to the Cu/Zn hybrid nanoparticles (Fig. 1B). However, 24 h of exposure inhibited all bacterial growth except for 100 µg/mL.

### Effect of Cu/Zn hybrid nanoparticles on bacterial spot severity and phytotoxicity under growth chamber conditions

**Figure 2:**
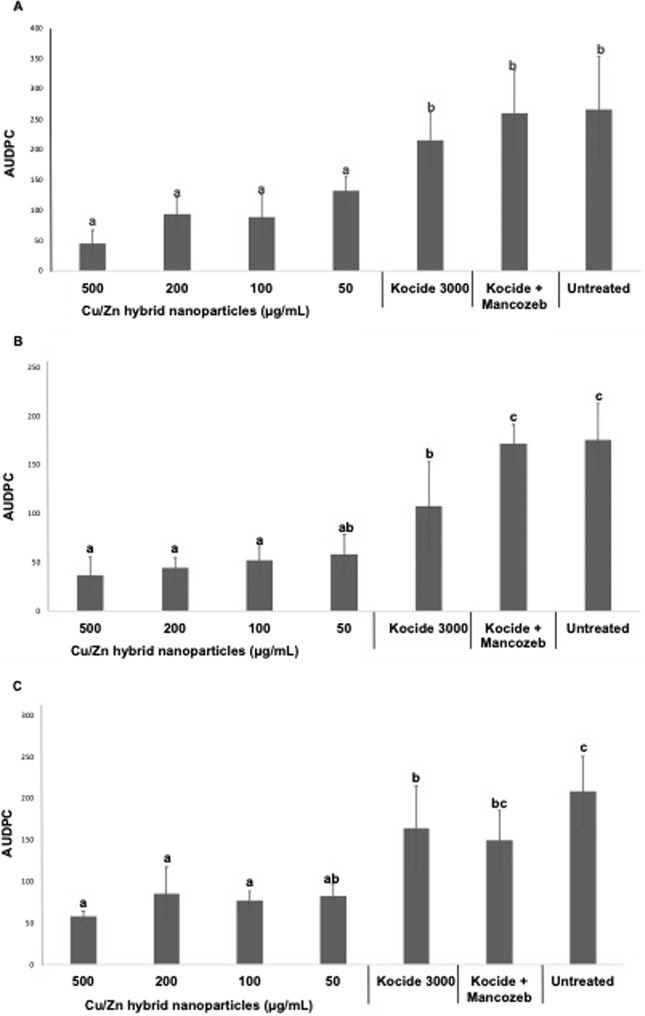
Effect of Cu/Zn hybrid nanoparticles on development of tomato bacterial spot *in planta* in growth chamber experiments. Error bars represent the standard deviation between four replicates. The area under disease progress curve (AUDPC) was calculated using Horsfall-Barratt scale collected every day since the third day after inoculation until twentieth day after inoculation. The experiments were performed three times (**A–C**).

Cu/Zn hybrid nanoparticles showed effectiveness for reducing bacterial spot disease severity as indicated by the AUDPC (Area under disease progress curve) in all experiments (Fig. 2). The results showed consistence between the replications and the repetitions.

Compared to untreated and plants treated with Kocide 3000 or Kocide 3000 amended with Mancozeb, Cu/Zn hybrid nanoparticles at 500, 200 and 100 µg/mL showed statistical difference dropping the severity of the disease. At the best performance, Cu/Zn at 500 µg/mL, dropped the AUDPC by up to 80% in comparison with untreated samples. When treated using the lowest concentration of the Cu/Zn hybrid nanoparticle (50 µg/mL) no significant differences were noted in reducing disease severity when compared to the controls. As expected, plants treated with Kocide and Kocide amended with Mancozeb, in general showed no statistical difference from the untreated control for controlling the disease.

**Figure 3:**
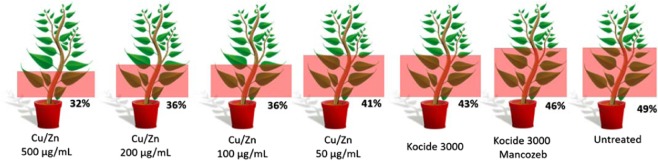
Percentage of leaves showing evident bacterial spot symptoms by the total number of leaves in the plant after application of Cu/Zn, Kocide 3000, Kocide 3000 amended by Mancozeb and untreated samples. Data collected after the last *in planta* experiment (20 days after inoculation).

Results for the evaluation of percentage of symptomatic leaves showed that when treated with Cu/Zn at 500 µg/mL the number of leaves showing evident lesions were 32% of the total plant against 49% for untreated samples (Fig. 3) The results were consistent for Cu/Zn 200 and 100 µg/mL where the percentage of symptomatic leaves were 36%. Again, samples treated with Cu/Zn at the lowest tested concentration (50 µg/mL) showed no significant differences in reduce the number of lesions when compared to the controls.

### Assessment of the impact of Cu/Zn hybrid nanoparticles on xanthomonadin, a carotenoid-like pigment in *Xanthomonas* spp

**Figure 4:**
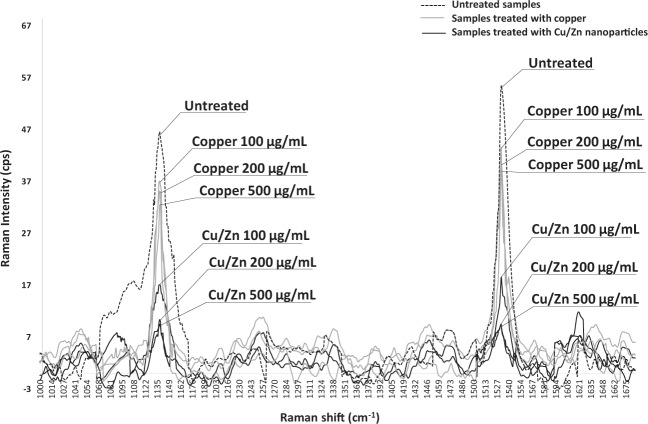
Raman intensity (cps) for copper-tolerant *Xanthomonas perforans* cells treated with Cu/Zn hybrid nanoparticles. The samples were prepared and treated using three different concentrations of Cu/Zn (500 µg/mL; 200 µg/mL and 100 µg/mL), three concentrations of Copper using Kocide 3000 (500 µg/mL; 200 µg/mL and 100 µg/mL), and one control treated with sterile tap water (untreated). After one-hour incubation, the spectra were collected using Raman spectroscope. The experiments were performed three times.

When exposed to Cu/Zn hybrid nanoparticles, the Raman intensity for the key peaks associated with xanthomonadin indicated by 1,138 and 1,532 cm^−1^ was significantly reduced in comparison with the untreated and copper treated samples (Fig. 4). Treatment with the lower concentration (100 µg /mL) of the Cu/Zn hybrid was as effective as the higher concentrations (200 µg/mL and 500 µg/mL) in decreasing the xanthomonadin levels in *X. perforans*. Samples treated with Kocide 3000 (Copper) had significantly less effect on xanthomonadin production in comparison with samples treated with Cu/Zn (Table 1).

**Table 1:** The effect of treatment of bacterial cells with Cu/Zn or copper at various concentrations on intensity shift in the Raman region that corresponds to Xanthomonadin presence in the bacterial cells.

Treatment (µg/mL)	Raman shift^z^ (cm^−1^)	Raman intensity^y^ (cps)	SEM^x^ (cps)
Cu/Zn (500)	1,138	9.81	±0.35
	1,532	9.43	±0.31
Cu/Zn (200)	1,138	10.80	±1.52
	1,532	9.65	±0.32
Cu/Zn (100)	1,138	17.30	±0.64
	1,532	18.48	±1.00
Copper (500) Kocide 3000	1,138	31.36	±2.28
	1,532	38.64	±1.02
Copper (200) Kocide 3000	1,138	32.54	±2.67
	1,532	40.55	±1.17
Copper (100) Kocide 3000	1,138	33.24	±3.40
	1,532	41.37	±2.00
Untreated	1,138	45.78	±0.87
	1,532	55.07	±0.43

The samples treated using Cu/Zn present less intensity hence less pigment presence in comparison with samples treated with Copper or untreated samples. The results are from samples exposed to chemicals for 1 hour.

^z^The Raman spectroscopic analysis is based on the shift presented by the movement of the molecules between energy states after absorption and release of energy. Xanthomonadin^34,41^ presents Raman shift at the spectral region at 1,138 and 1,532 cm^−1^.

^y^The Raman signals are measured in counts per second (cps) showing the Raman intensity correlated with the time of laser incidence in the sample.

^x^The experiments were performed 4 times and SEM indicates standard error of mean between the replicates.

## Discussion

When developing novel nanoparticles for bacterial disease management, various factors need to be considered. This includes ability of the nanoparticle to cause rapid bacterial cell inactivation, effectiveness in reducing disease severity on plants, and no phytoxicity from application on plants that can generate barriers for its further development and commercialization. In this study we demonstrated for the first time the potential of a hybrid nanoparticle (Cu/Zn) in managing bacterial spot of tomato incited by copper-tolerant *X. perforans*. Cu/Zn hybrid nanoparticle showed antibacterial activity against *X. perforans* even at the lowest tested concentration, and completely eliminated the copper-tolerant strain within 15 min of exposure. In greenhouse experiments the Cu/Zn hybrid nanoparticle at concentrations above 50 µg/mL unlike copper-mancozeb or copper alone consistently controlled disease compared to the untreated control. Equally, important to the effectiveness of a new approach in disease management is the lack of any phytotoxicity to plants by Cu/Zn hybrid nanoparticle application. The results of this study suggest that field studies will be needed to investigate whether Cu/Zn hybrid nanoparticle can provide efficacy at low rates of Cu and Zn. Even though other studies have shown the potential of Zn based nanoparticles^27^ and micron-sized particles^29^ to inactivate bacterial growth, one of this study^29^ also suggests potential for fast development of resistance to Zn in the bacterium upon regular field applications. Field studies will provide further understanding on this risk factor.

Another factor that should be considered in the development of a new compound is its mode of action. Morphological changes in bacterial cells such as membrane integrity or physical structure of the cells when analyzed by transmission electron microscopy, confocal microscopy or various other techniques can provide useful information in understanding the mode of action. Among the techniques, Raman spectroscopy is a useful approach for assessing biochemical alterations in bacterial cells. The pathogen studied in this research, *Xanthomonas perforans*, is well known in producing xanthomonadin. Xanthomonadin is a specific and unique pigment produced by bacterial strains in the genus Xanthomonas. These pigments are brominated, aryl-polyene, yellow, water-insoluble that are associated exclusively with the other membrane of the bacterial cell wall and produced by all yellow colored *Xanthomonas* spp.^36^. The pigment is critical as a survival mechanism for the bacteria against photobiological damage. Studies examining the biological role of xanthomonadin utilizing xanthomonadin mutant strains showed that the mutants were significantly affected in its epiphytic survival and host infection via plant hydathodes^38^. This current study demonstrates for the first time the inhibitory properties of Cu/Zn hybrid nanoparticle on xanthomonadin in *X. perforans*. Interestingly, it appears that conventional micron-sized copper bactericides have a different mode of action than the Cu/Zn hybrid nanoparticles given that the former has a minimal effect on xanthomonadin whereas Cu/Zn nanoparticles have a dramatic effect as determined by Raman spectroscopy. Additional studies are needed in the future to further understand the mechanism of action by which Cu/Zn hybrid nanoparticles are affecting xanthomonadin, and other components of the bacterial cell wall in comparison to other chemical compounds.

In order to reduce the probability for bacterial cells developing resistance to nanoparticles, it is necessary to design a multipronged approach for using bactericides with different modes of action on bacterial pathogens. This can be achieved in part by understanding the structural and biochemical changes on bacterial cells by nanoparticle exposure. Epifluorescence microscopy has previously provided insight into whether the nano materials are bactericidal or bacteriostatic in nature^26^. Scanning electron microscopy (SEM) and transmission electron microscopy (TEM) have been shown to provide information on structural modifications on the surface and interior of bacterial cells upon exposure to the Cu/Zn hybrid nanoparticles^26^. Finally, Surface Enhanced Raman spectroscopy (SERS) and Resonance Raman Spectroscopy can provide further in-depth information on biochemical changes to the bacterial cells upon exposure to Cu/Zn hybrid nanoparticles in addition to the effects noted on xanthomonadin in this study.

Nanoparticles with apparently different mechanisms of action have been demonstrated to perform better than standard bactericides for management of copper tolerant *Xanthomonas* spp. strains. For example, Paret *et al*.^23^ determined that TiO_2_/Zn had high photocatalytic activity against *X perforans in vitro* as well as for control of bacterial spot in greenhouse and field experiments without negatively affecting yield. However, the effective application rate of TiO_2_/Zn was at >500 ppm. Application of large amounts of chemicals can increase cost of production and impacts on environment and lead to occupational safety concerns. In another study by Strayer *et al*.^8^, three copper composites and showed that they perform well in greenhouse experiments and these findings translated well into field scale studies with efficacy in reducing bacterial spot disease severity at Cu levels at 100 µg/mL.

At the same importance of mode of action understanding, element behavior such as plant absorption and measurements of chemical attachment on leaf surface are also important assessments needed due to the importance in understanding toxicological risks, soil and microbiome impacts and best practices of applications. Many tools in analytical chemistry can be used to assess the elemental composition in the plant surface and environment. For example, traces of elemental ions can be measured using X-ray fluorescence as tested by Turner *et al*.^39^. They measured traces of As, Cu, Pb and Zn on leaf surfaces using a field portable x-ray fluorescence equipment (FP-XRF). Among other examples, Inductively Coupled Plasma Optical Emission Spectroscopy (ICP-OES) or Mass Spectrometry (ICP/MS) can be used to measure the ionic deposition on leaf surfaces as well as fruit or soil contamination by chemicals after applications^26^. For example, research conducted by Liao *et al*.^26^ evaluated, elements in tomato fruits after MgO nanoparticle applications in comparison with conventional Cu and water controls. The research used ICP-OES to analyze fruit and peels for the accumulation of Al, B, Ca, Cu, Fe, K, Mg, Mo, Na, P, S and Zn. This research showed that fruits exposed to multiple applications of MgO nanoparticles, did not had statistical difference in the elements accumulation when compared with untreated fruits. Similar studies described above if conducted using Cu/Zn hybrid could help in further understanding the toxicological risks associated with this nanoparticle if used under field conditions. The studies described above could shed more light on the mechanism(s) of action of Cu/Zn hybrid on *X. perforans* cells and *in planta*.

Given the difficulties of controlling bacterial spot with conventional bactericides, in which regular weekly applications and, in some cases, up to 18–20 applications in a single season with primarily copper-based bactericides, which are ineffective, it has been a focus of our research to develop alternative strategies using nanoparticle technology^8,24–26^. Thus, development of hybrid nanoparticles like Cu/Zn offers the use of multiple elements that could potentially provide multi-site mode of action on bacterial pathogens, and hence be more effective than existing single element conventional Cu alone used in existing bactericides. Furthermore, there is a possibility that the use of multiple elements would reduce the likelihood for bacteria to develop resistance.

In summary, the results of this study demonstrate the antibacterial activity of hybrid Cu/Zn nanoparticles on the destructive copper-tolerant bacterial spot pathogen, *X. perforans*. Cu/Zn directly affected the production of the biologically relevant pigment, xanthomonadin in the bacterial cell membrane of *Xanthomonas* spp. Future experiments using the hybrid Cu/Zn as an active ingredient formulated for field applications are required to evaluate the impact of the nano material when applied periodically on disease management, phytotoxicity, crop yield, fruit elemental accumulation and microbiome impacts in comparison to conventional Cu bactericides.

## Materials and Methods

### *In vitro* studies to evaluate antibacterial activity of Cu/Zn hybrid nanoparticles on copper-tolerant and copper-sensitive *Xanthomonas perforans* strains

Copper-tolerant *X. perforans* strain, GEV485, and copper-sensitive *X. perforans* strain, 91–118, were used to evaluate antimicrobial activity of Cu/Zn hybrid nanoparticles *in vitro*. The bacterial strains were streaked for inoculum on nutrient agar medium (NA; Difco^TM^ Sparks, MD) and incubated at 28 °C for 24 h. For culturing of GEV485, copper (II) sulfate pentahydrate (CuSO_4_ 5H_2_O) (Fisher Scientific – Hampton, NH) was amended, at 20 ppm, to nutrient agar serving as copper tolerance selective agent. Bacterial cells were collected from NA plates, re-suspended in sterile deionized water, and adjusted to A_600_ = 0.3 at λ = 600 nm (~5 × 10^8^ CFU/mL). The final concentration of each suspension was adjusted to 10^5^ CFU/mL, and 20 µL of each suspension was added to tubes containing different concentrations of Cu/Zn hybrid nanoparticles ((US Nano, Houston, TX) Cu/Zn; 99.9% purity, 50:50% proportion 40 nm–100 nm). at 1,000; 500; 200; and 100 µg/mL. The element was adding to sterile tap water and dispersed using ultrasonic bath for 5 minutes at 20 kHz. Controls included tubes containing 2 mL of copper suspension at same concentrations of test compounds and tubes containing 2 mL of sterilized tap water served as untreated control. Copper treatment tubes were prepared using Kocide 3000 (DuPont, Wilmington DE) that contains 30% metallic Cu in the form of copper hydroxide (Cu(OH)_2_). All tubes were incubated at 28 °C on an orbital shaker at 150 rpm for the following times: 15 min, 1 h, 4 h, and 24 h. After incubation, 50 µL from each tube was plated on NA medium and incubated at 28 °C for 48 h. After incubation, total viable counts for each tube were counted. Each treatment consisted of three replicates and the experiment was performed two times.

### *In planta* studies to evaluate the phytotoxicity and potential of Cu/Zn nanoparticles in reducing bacterial spot disease severity

Four-week old FL 47 (Seminis Vegetable Seed, St Louis, MO) tomato plants, grown in seedling trays and transplanted to larger pots, were moved to a growth chamber with controlled conditions (28 °C, 12 h photoperiod, and 80% relative humidity). After three days, the plants were subjected to foliar sprays: four treatments of Cu/Zn hybrid nanoparticles, Kocide 3000, and Kocide 3000 + Mancozeb (Manzate 75DF, Griffin Corporation, Valdosta, GA). Treatments were prepared at the following concentrations: 500, 200, 100, and 50 µg/mL. The untreated control consisted of four plants being sprayed with sterilized water. After application, leaf surfaces were allowed to air dry for 4 h. Afterwards, both abaxial and adaxial leaf surfaces were inoculated by spraying with a bacterial suspension (10^8^ CFU/mL) of *X. perforans* GEV485. Following inoculation plants were immediately covered with plastic bags to ensure high humidity and effective bacterial inoculation. Plastic bags were removed three days post inoculation, and plants remained in the growth chamber under the same conditions. Disease severity and phytotoxicity were evaluated daily using the Horsfall-Barratt scale^40^, starting from three days up to twenty days post infection. Three experiments were performed, and each treatment consisted of four replicates.

For the last round of *in planta* experiments, after 20 days, the plants were evaluated for the numbers of symptomatic leaves. For the evaluation, the percentages of leaves showing lesions were counted by the total numbers of leaves in the plant.

### Near-Infra Red Raman spectroscopy studies to elucidate changes in xanthomonadin of *X. perforans* following exposure to Cu/Zn hybrid nanoparticles and commercial Cu

Fresh bacterial inoculum of the copper-tolerant strain GEV485 was used to create a suspension with a final concentration of 10^8^ CFU/mL. Five hundred microliters of the suspension was added to 2 mL of Cu/Zn hybrid nanoparticle suspensions at 500, 200, and 100 µg/mL). As a negative control, 500 µL of suspension was added to 2 mL of sterilized water. All tubes were incubated at 28 °C in an orbital shaker at 150 rpm for 1 h. After incubation, 1 mL of the sample was transferred to a microfuge tube and centrifuged for 3 min at 14,000 rpm. Supernatant was discarded and 1 mL of NaCl 0.85% was added to the pellet. The samples were centrifuged once more under the same conditions and then washed two times using sterilized water. One hundred microliters of sterilized water were then added to the final sample, then the samples were filtered through micropore filter (4 µm) to clean the sample off for any chemical remaining in the sample. Glass microscope slides were covered with aluminum foil and cleaned using 70% ethanol. Five microliter aliquots of the samples were placed on slides and allowed to air dry under a desk lamp for 20 min. After drying, slides were analyzed by Raman spectroscopy. Raman spectroscope (ThermoFisher Scientific, Waltham, MA), laser wavelength was set at 785 nm, and the equipment was configured to take spectra with a laser power of 20 mW, aperture of 25 µm slit, collect exposure at 60 s, and sample exposure at four times for each spectra. The spectral data were analyzed using OMNIC (ThermoFisher Scientific, Waltham, MA). Four spectra were taken for each treatment followed by the substrate subtraction, automatic baseline correction and smooth correction as described by Paret *et al*.^41^.

### Statistical analysis

Statistical analysis was conducted using IBM SPSS Statistics software (version 22; Armonk, NY). Significantly differences between groups were calculated using the post-hoc test method Student-Newman-Keuls (SNK) with a *P value* of 0.05. For *in vitro* assays, the log populations were calculated and the data were organized by treatment, concentration and exposure period. *In planta* data were collected and organized by the treatment and concentration. The data collected for both *in vitro* and *in planta* experiments were analyzed and the results were compared to ensure consistency between the repetitions.
